# Multi-Functional Luminescent Coating for Wood Fabric Based on Silica Sol-Gel Approach

**DOI:** 10.3390/polym13010127

**Published:** 2020-12-30

**Authors:** Ming Li, Rui Huang, Zhengquan Fu, Di Wang, Chengyu Wang, Jian Li

**Affiliations:** 1 Engineering Research Center of Advanced Wooden Materials (Ministry of Education), Northeast Forestry University, Harbin 150040, China; alexlavie@outlook.com (M.L.); huangrui9759@163.com (R.H.); fzq@nefu.edu.cn (Z.F.); wangcy@nefu.edu.cn (C.W.); nefujianli@163.com (J.L.); 2 Key Laboratory of Bio-Based Material Science and Technology (Ministry of Education), Northeast Forestry University, Harbin 150040, China; 3 Collage of Material Science & Engineering, Northeast Forestry University, Harbin 150040, China

**Keywords:** photoluminescence, thermal stability, optical properties, anti-ultraviolet aging properties

## Abstract

Environmentally friendly protection coatings have obtained increasing attention for their use in wooden materials, which can be destroyed easily when exposed to outdoor environments. A series of silane sol coatings coordinated with Eu^3+^ was prepared by hydrolyzing silane compounds. The obtained luminescent coating with three-dimensional net structure showed excellent optical, anti-ultraviolet aging, and thermal stability. The hybrid silane-modified compound coating was well-distributed on the wood by Si–O bonds to prevent its removal. The compound coating could stave off the decomposition of wood by converting ultraviolet light into red light and a charring action can endow the wood with thermal stability at high temperature, demonstrating the improvement of fire resistance and radiation residence following prolonged exposure to ultraviolet light, proving its excellent anti-ultraviolet aging properties.

## 1. Introduction

Wood is a natural material mostly composed of cellulose, lignin, and hemicellulose, which is uneven distributed in the cell and intercellular layer [1,2,3,4]. Therefore, the wood contains a large number of hydroxyl groups, which are susceptible to water to cause the damage of the wood. What is more, abundant biological characteristics will also make it vulnerable to attacks by organisms and microorganisms in the environment, affecting its stability and applications [5]. Over the years, in the development and utilization of wood products, outdoor use of wood products gradually increased wood surface in the surrounding environment. In recent years, wood products used outdoors have gradually increased. Functional modification of wooden composites has long been a common trend because of their wide applications in homes, heating, furniture, and paper [6]. However, wooden materials easily decompose when exposed with solar radiation and vapor in the air under normal temperature conditions. Thus, it is of great significance to develop sustainable and cost-effective coatings to achieve thermal stability and anti-ultraviolet properties.

Although the luminescence efficiency of rare earth are so low due to their parity forbidden 4f-4f transitions, the drawback could be conquered through the introduction of the ligand to form the rare earth complex [7]. The luminescence process of rare-earth complexes can be describe as an energy transfer process that after the formation of singlet excited state molecules attributed to energy absorption through the irradiation of excitation light, the singlet excited state energy is transferred to the triplet state by non-radiative transition, next to which the triplet energy is transferred to the rare earth ions’ excited state without radiation together with the radiation process from the excited state of metal ions to ground state resulting in light emission. This process of absorbing energy from the ligand and then transferring the energy to the rare earth ions is also called the antenna effect [8]. Rare earth complexes are widely used because of their narrow spectral band, high luminous efficiency, and strong color purity [9].

The degree of aging of the wood composite material depends on the sensitivity of the surface of the material to sunlight, which is related to the chemical structure of the material. Due to the refraction and scattering of light, only a small part of the sunlight shines on the ground, and its wavelength is approximately between 290–430 nm, and the sunlight that negatively affects the surface of the material. Ultraviolet (UV) light irradiation in sunlight can mostly degrade the structure of wood and generate great amount of free radicals, a phenomenon that is mostly attributed to the lignin in the wood, which is so sensitive that it can react with oxygen under certain conditions outdoors to produce chromophoric carbonyl and carboxyl groups, leading to the color change of the wood [10,11,12,13,14]. Traditional organic coatings inevitably decompose when exposed to ultraviolet light for a long time [15]. Functional treatment of wooden materials has long been a major issue because of their massive application in everyday life and high hygroscopicity and decomposition which make their service life greatly shorten.

Organosilicon, a kind of synthetic polymer, whose main chain skeleton is formed by alternating oxygen and silicon atoms connected with other organic segments or functional groups, has been widely used in various fields, including construction, packaging, transportation, medical equipment, and other industries [16,17,18,19]. It has become an indispensable type of new polymer material used in daily life and has a superior high temperature resistance performance and anti-ultraviolet aging properties [20,21]. Moreover, to strengthen these anti-ultraviolet aging properties, we introduce rare earth ions dipped in coatings in order to form a coordinated environment to convert the UV light absorbed by photosensitive chromophores into harmless red-light emission by Eu^3+^ in order to prevent the decomposition process [22]. In addition, the coordination of Eu^3+^ ions with −C=O groups of the ligand will increase the degree of crosslinking of the coating network [23,24].

**Scheme 1:**
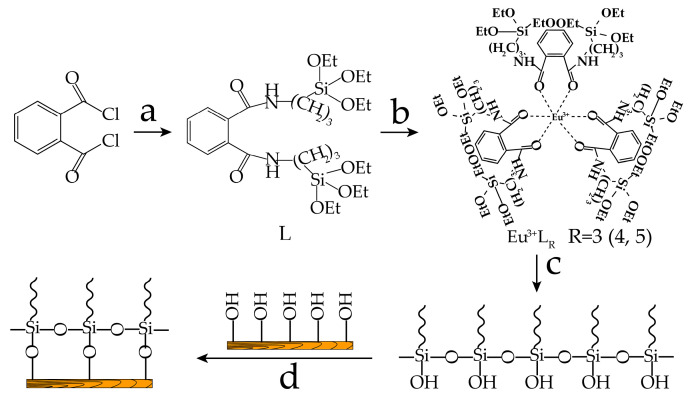
Synthetic routes to coating composites. (**a**) aminopropyl trimethoxy silane/CH_2_Cl_2_/room temperature, 2 h (**b**) Eu (NO_3_)_3_ 6H_2_O/C_2_H_5_OH/80 °C, 8 h (**c**) HCl/H_2_O/C_2_H_5_OH/40–60 °C, 12 h (**d**) room temperature, 24 h/85 °C, 2 h.

Herein, multi-functional luminescent protection coatings were prepared by hydrolysis and condensation reactions among the modified silane monomers coordinated with europium (III) ions. The coating composite was obtained through a condensation reaction between the silicon hydroxyl groups in the coating and the surface hydroxyl group of the wood surface as shown in Scheme 1. Mass spectra were measured using electrospray ionization mass spectra (ESI-MS) in the positive mode from tetrahydrofuran solutions. The surface morphology structures were explored by scanning electron microscopy (SEM), Energy Dispersive X-ray Analysis (EDXA), and FTIR spectroscopy (FTIR). Derivative thermogravimetry (DTG) and thermogravimetry (TG) analysis is used to certify the pyrolysis reactions of coated wooden composites from room temperature to 800 °C to investigate their thermal stability. In addition, the anti-ultraviolet aging ability of the coating composites was also predicted. 

## 2. Materials and Methods 

### 2.1. Materials

Poplar wood slices with the sizes of 10 mm (longitudinal) × 5 mm (tangential) × 2 mm (radial) were ultrasonically washed in acetone, ethanol and deionized water three times for 10 min in turn and dried at 50 °C for 48 h in a vacuum. Europium nitrate hexahydrate (Eu (NO_3_)_3_·6H_2_O) (99.99%) and aminopropyl trimethoxy silane (99%) used throughout the research were provide by Beijing Huaweiruike Co., Ltd., Beijing, China. Ethanol (99.7%) and phthaloyl chloride (98%) were supplied by Tianjin Kaitong Chemical Reagent Co., Ltd. (Tianjin, China) without further purification.

### 2.2. Synthesis of Modified Silane Monomer

Phthaloyl chloride (0.005 mol) in methylene chloride (10 mL) was added to the solution of aminopropyl trimethoxy silane (0.01 mol) in methylene chloride (10 mL) at 0 °C. Then, the mixture was stirred at room temperature for 2 h, after which the solution were put into a vacuum at 45 °C to remove the solvent. At last, the colorless and transparent product was obtained 

### 2.3. Synthesis of Europium Coordination Complex

Eu (NO_3_)_3_·6H_2_O and modified silane monomer were accurately weighed with different molar ratios (1:3, 1:4, 1:5) and dissolved in 10 mL of anhydrous ethanol. Then, the mixture was heated continuously under reflux at 80 °C for 8 h to obtain the complex solution. 

### 2.4. Synthesis of Organosilicon Sol Coordinated by Europium (III)

Eu (NO_3_)_3_·6H_2_O and modified silane monomer were accurately weighed with different molar ratios (1:3, 1:4, 1:5) and dissolved in 10 mL of anhydrous ethanol, respectively. Then, the mixture was heated continuously under reflux at 80 °C for 8 h. Finally, the mixture was diluted with ethanol for concentrations of 0.08 mol/L. The appropriate amount of distilled water and 4–8 drops of 1mol/L dilute hydrochloric acid were added to the complex’s solution above and stirring magnetically in a water bath (40 °C, 45 °C, 50 °C, 55 °C, 60 °C) with reflux condensation and stirring for 12 h to obtain the sol coatings. 

### 2.5. Synthesis of Coating Composites 

The pre-treated woods were immersed into the 20 mL of sol coatings at room temperatures under continuous mechanical stirring for 24 h and a layer of Si–O–Si network structure covered on wood surface by Si–O covalent bond. Then, the samples were dried at 85 °C for over 2 h in the vacuum. Finally, the coating composites were obtained.

### 2.6. Artificial Ultraviolet Aging Test

Various samples simulated ultraviolet aging under ultraviolet light of high energy in the same conditions to investigate the sol coating composites with radiation time ranging from 0 to 20 d. During the color change test, the color variation of the coating composites surface before and after the UV irradiation were measured with CIELAB system in accordance with the ISO-2470 standard. CIELAB L*, a*, b*, parameters were measured at 10 locations on each specimen and every sample had three replicas.

ΔL* = L1* − L0*(1)

Δa* = a1* − a0* (2)

Δb* = b1* − b0* (3)

where ΔL*, Δa*, and Δb* values were the difference of the final (L1*, a1*, b1*) and initial (L0*, a0*, b0*) color values after and before UV irradiation, respectively. 

The values above were used to obtain the overall color change parameter ΔE* as a function of the weathering time:

ΔE* = [(ΔL*)^2^ + (Δa*)^2^ + (Δb*)^2^]^1/2^ (4)

A small ΔE* value was corresponded to a subtle color difference indicating strong resistance of coating composite to UV radiation.

## 3. Results

### 3.1. Structural Characteristics

**Figure 1:**
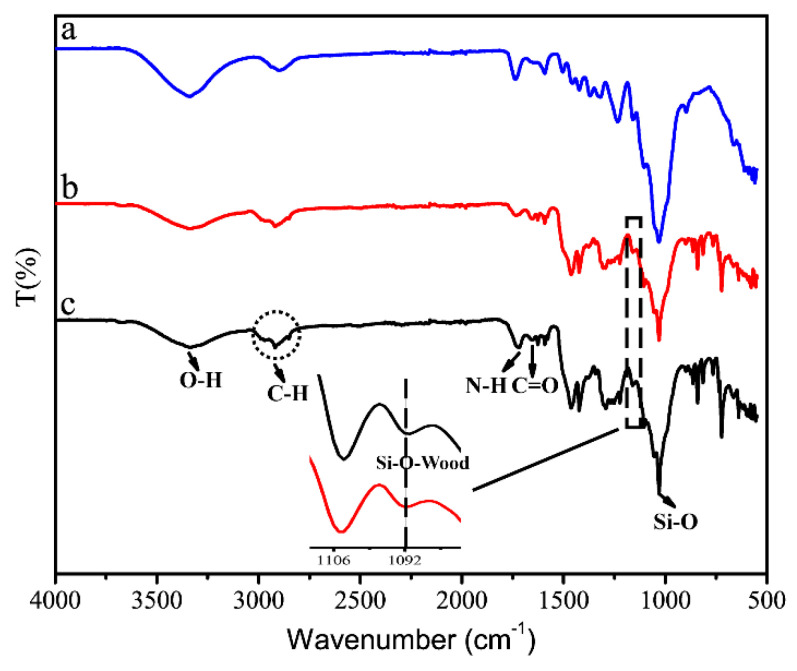
FT-IR spectra for (**a**) the untreated wood, (**b**) the sol coating composite, (**c**) the sol coating composite under UV-light radiation (340 nm) for 480 h.

FTIR spectra of the untreated wood, the coating wood, and the coating composite under UV light radiation for 480 h was shown in Figure 1. For the untreated wood sample, the peak appears at 3336 cm^−1^ was assigned to the stretching vibration of the O–H stretching groups [25,26]. After modification, the intensity of broad absorption peak appeared at 3336 cm^−1^ became weak, which indicated that a condensation reaction between the silanol groups of hydrolyzed aminopropyl trimethoxy silane and the surface hydroxyl group of the wood surface had occurred [27]. The absorption band at 2913 cm^−1^ corresponds to the C–H stretching vibrations. In addition, after the surface was covered by the sol coating, new adsorption bands appear at 1657 cm^−1^ and 1730 cm^−1^, which is attributed to C=O stretching vibrations and N–H bending vibration, respectively [28], proving the existence of a secondary amide group on the coating surface. The peaks around 1025 cm^−1^ corresponded to Si–O–Si and Si–O-C stretching vibrations after coating [29]. The absorption peak at 1092 cm^−1^ was ascribed to the Si–O–wood bond, indicating a strong polycondensation grafting reaction between silanol groups and the wood surface [30]. More importantly, the curves of the wood after a long period of radiation exposure exhibited similar behavior in relation to their FTIR spectra compared with the coating composite, which proves the ability of the sol coating to protect wood from ultraviolet light damage.

The ESI-MS spectra of organosilicon sol coordinated by europium (III) in the positive mode are shown in Figures S1 and S2 in the supplementary material. The spectra exhibit peaks at m/z = 630.05 and 721.07 that are assigned to the ligands (see Supplemental Information, Figure S1 in the supplementary material), verifying the formation of Si–O structure. In addition, several fragments containing europium were observed in the mass spectra (Table S1 in the supplementary material).

### 3.2. SEM-EDX Analysis

**Figure 2:**
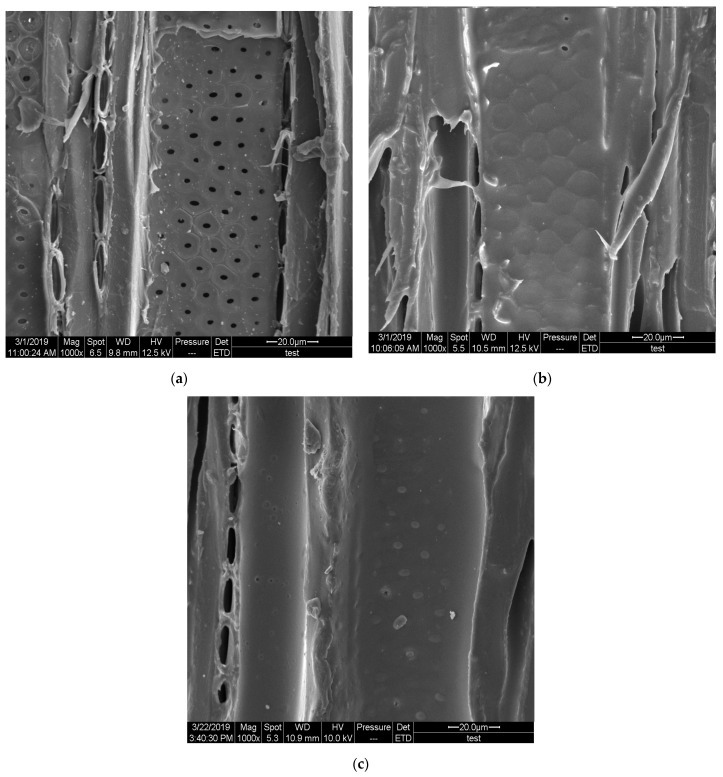
SEM spectra of (**a**) the untreated wood, (**b**) the sol coating composite, (**c**) and the sol coating composite under UV light radiation (340 nm) for 480 h.

**Figure 3:**
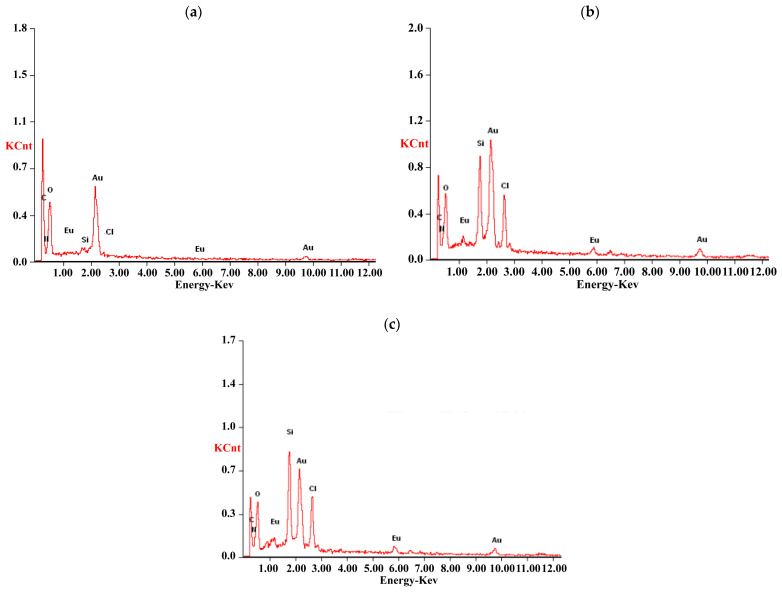
Energy Dispersive X-ray Analysis (EDXA) spectra of (**a**) the untreated wood, (**b**) the sol coating composite, (**c**) and the sol coating composite under UV light radiation (340 nm) for 480 h.

The SEM results in Figure 2 exhibit the surface morphologies of the untreated wood, the sol coating composite, and the sol coating composite under UV light radiation (340 nm) for 480 h. In Figure 2a, the pristine tracheid of the wood is smooth. A homogeneous and continuous network structure masks the surface (Figure 2b), which is attributed to the sol–gel reaction on the silane, indicating that the sol coatings were effectively applied to the wood surface. The chemical elements on the surface were explored using their EDXA spectra, as shown in Figure 3. Before treating the wood, there are only carbon, oxygen, nitrogen, and gold elements that could be detected. After coating, silicon, europium, and chloride could be found from the spectra due to the introduction of the sol coating. The element distribution of the coating composite changed slightly under UV radiation over the 480 h, from which it could be predicted that part of the coating was subject to strong radiation during its protection of the wood surface. As shown in Figure 2c, after a long period of radiation exposure, the coating structure protecting the wood partially disappeared.

### 3.3. Thermal Stability

**Figure 4:**
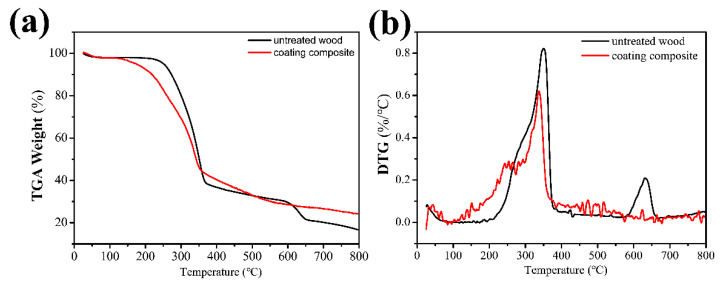
TGA curves (**a**) and DTG curves (**b**) for the untreated wood and coating composite.

For untreated wood, there are four thermal degradation steps that have been clearly observed in the N_2_ atmosphere (Figure 4). The first step occurred below 120 °C and the subtle weight loss could be attributed to the loss of water. The most weight loss happened in the second region from 120 to 400 °C, and was caused by the depolymerization of the hemicellulose, giving rise to D-xylose as well as to some oligosaccharide mixtures. Then, further oxidation of the lignin took place at a higher temperature [31,32]. Compared with the untreated wood, as shown in Figure 4 and Table 1, the introduction of the sol coating increased the decomposition process of the wood with lower T_max2_ values, which could be attributed to the stable thermal silicide layer initially generated by the reaction of hydroxyl and silane hydroxyl. The initial degradation was conducive to improving the thermal stability of the wood as stable thermal silicious structures formed, which could postpone the next stage of its degradation. Although the thermal stability of the coating on the wood was reduced at the beginning, it was significantly strengthened in the last decomposition step, which could also be indicated by the elevated T_max3_ value and increased residue from 600 to 800 °C.

### 3.4. Aging Property and Optical Stability 

**Figure 5:**
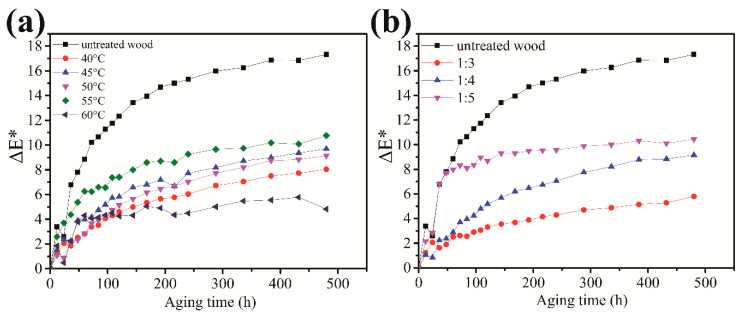
Variation tendency of ΔE* of the untreated wood and coating composite with sol coatings obtained under (**a**) different reaction temperatures (40 °C, 45 °C, 50 °C, 55 °C, 60 °C) (**b**) different ratio between Eu^3+^ and ligands.

The color variations during the UV light (340 nm) radiation were measured via accelerated aging tests to explore the anti-ultraviolet property of the wood as well as the coating composite. UV aging resistance was predicted by the ΔE* before and after UV radiation. It can be seen that the ΔE* value of the untreated wood is higher than the coating composite. Figure 5a shows the trends of the ΔE* in the samples with sol coatings obtained under different reaction temperatures (40 °C, 45 °C, 50 °C, 55 °C, 60 °C) before and after UV light radiation. The ΔE* of the sample coated with the sol coating obtained at 60 °C is the lowest, predicting that the UV aging resistance of the sample is proportional to the reaction temperature of the sol coating. The photochromic performance of the coating composite becomes gradually stable with the UV exposure time increasing. The wood with coatings obtained at 60 °C exhibited a stronger UV resistance ability. As an important indicator of the service life of the material, the ΔE* values play a necessary part. 

Figure 5b shows the ΔE* of the untreated wood and coating composite under UV light radiation in relation to the variability of the different ratios between Eu^3+^ and ligands. After 480 h of UV light radiation, the ΔE* values of all coating composites exhibited no significant fluctuations and only slight variations, indicating the good photochromic stability of the sol coating. A ratio of 1:3 showed the best UV light resistance with less color variation. Meanwhile, the largest ratio for the changing values of natural wood and modified wood is 6.29, which belongs to the ratio 1:3 and approaches two times that of the ratio 1:4. The ratio between Eu^3+^ and the ligands of 1:3 was the most stable after 100 h of aging for this variable.

**Figure 6:**
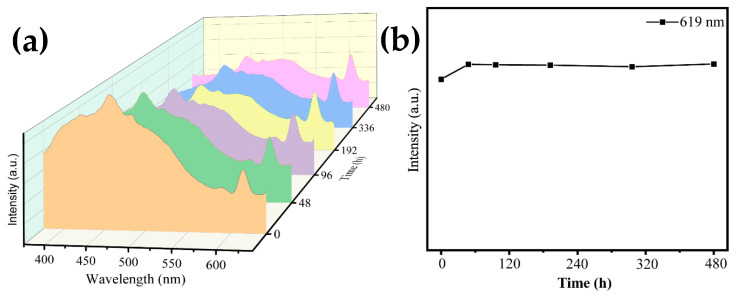
(**a**) Emission spectra of coating composite during the radiation of UV-light for different time (0 h, 48 h, 96 h, 192 h, 336 h, 480 h) and (**b**) variation tendency of emission intensity (614 nm).

To further investigate the optical properties of the coating composite under sustained UV radiation (340 nm), the variations in the values of the emission peaks of Eu^3+^ (614 nm) in the coating composites obtained at the most optional conditions with a ratio of 1:3 at 60 °C were evaluated. As shown in Figure 6, it can be seen that, after ultraviolet radiation, the fluorescence of wood is enhanced, which is perhaps due to the fact that, after a long period of radiation exposure, the silane undergoes further hydrolysis and condensation reactions, leading to the formation of an Si–O network structure, which will increase the rigidity of the ligand and reduce the energy loss by non-radiative deactivation [33]. On the contrary, the condensation reaction of organic–inorganic hybrid coatings change the coordination environment of Eu^3+^, which could be reflected by the ratio of ^5^D_0_→^7^F_2_ (614 nm) transition to ^5^D_0_→^7^F_1_ (590 nm) transition [34,35,36]. The larger ratio of ^5^D_0_→^7^F_2_ transition to ^5^D_0_→^7^F_1_ means that the chemical environment symmetry around the Eu^3+^ ion is decreased [37,38]. From Table 2, it can be seen that, after 14 days of radiation, the ratio increased from 1.40 to 1.74, then decreased to 1.68, which may be attributed to the fact that, initially, the silane coating was cross-linked to form an Si–O network structure under ultraviolet light radiation. However, the layer structure is destroyed during long-term radiation exposure, and the coordination environment of the rare earth ions is changed back to its original state, only with a higher symmetry. From Figure 6, it can be indicated that the coating composites we prepared have an excellent fluorescence stability, and can transfer UV light to red light under long-term radiation exposure in order to protect the surface of wood from intense radiation.

## 4. Conclusions

The prepared organic–inorganic hybrid silica coatings, based on natural wood materials, effectively improved the thermal stability and anti-ultraviolet aging ability of wood. Via TG analysis, the coated wood with the hybrid silica coatings was found to be able to extinguish flames, and the texture and structure of the residual chars was maintained, demonstrating the good efficiency of the coatings on wood at high temperatures, due to the formation of the Si–O network, suggesting an evident intumescent charring mechanism for the coated woods. The coating composites also exhibit a better durability during 480 hours’ radiation exposure using UV light (340 nm), which could be attribute to the fact that the silane coating was cross-linked to form an Si–O network structure under ultraviolet light irradiation. More importantly, the formation of the Si–O network structure increases the rigidity of the ligand and reduces the energy loss by non-radiative deactivation. Moreover, the coordination environment of the rare earth ions was changed back to its original state, only with a higher symmetry, which could enhance the energy transfer from the ligand to the rare earth ions in order to convert ultraviolet light into red light efficiently and reduce the damage caused by ultraviolet radiation on the coating composite, leading to a higher fluorescence stability. These hybrid silica coatings could therefore be considered as an effective and environmentally sustainable approach for the protection of wood.

## Figures and Tables

**Table 1:** Results of thermogravimetric analysis.

Sample	T_max1_ (°C)	Residue at 120 °C (%)	T_max2_ (°C)	Residue at 400 °C (%)	T_max3_ (°C)	Residue at 600 °C (%)	T_max4_ (°C)	Residue at 700 °C (%)	T_max5_ (°C)	Residue at 800 °C (%)
Untreated wood	69.2	98.0	370.7	36.8	596.8	29.6	651.4	20.2	800	16.7
Coating composite	72.2	97.7	354.3	40.3	573.1	28.6	--	26.6	800	24.2

**Table 2:** The ratio of ^5^D_0_→^7^F_2_/^5^D_0_→^7^F_1_ under different time radiation.

Radiation Time (d)	0	2	4	8	14	20
^5^D_0_→^7^F_2_/^5^D_0_→^7^F_1_	1.40	1.51	1.62	1.70	1.74	1.68

## Supplementary Materials

The following are available online at https://www.mdpi.com/2073-4360/13/1/127/s1, Figure S1: ESI-MS positive mode of organosilicon sol coordinated by europium (III) dissolved in THF showing the corresponding fragment inset, Figure S2: Geometry structures of products, Table S1: Fragments assignments according to the experimental data of organosilicon sol coordinated by europium (III).
