# Supplementary material for: Multi-Functional Luminescent Coating for Wood Fabric Based on Silica Sol-Gel Approach
Source: Polymers (Basel). 2020 Dec 30;13(1):127. doi: 10.3390/polym13010127 (PMC7795870; doi:10.3390/polym13010127)
Supplement: Supplementary file 1 [file polymers-13-00127-s001.pdf]

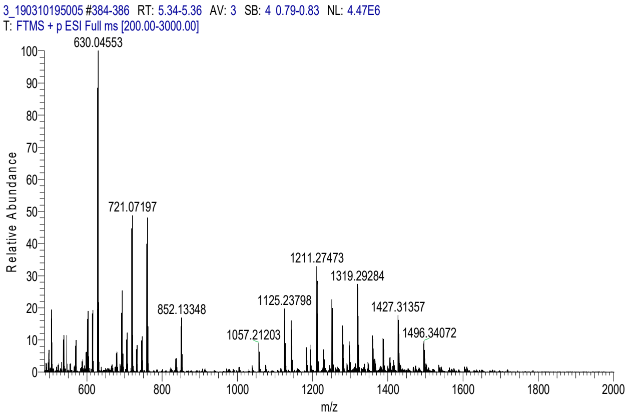

Figure S1. ESI-MS positive mode of organosilicon sol coordinated by europium (III) dissolved in THF showing the corresponding fragment inset

Table S1. Fragments assignments according to the experimental data of organosilicon sol coordinated by europium (III)

Sample	Calculated Value (m/z)	Experimental Value (m/z)	Formula Structure
1	630.890	630.046	$[Eu(C_{14}H_{18}O_5N_2Si_2Cl_3+Na)]^+$
2	722.980	721.072	$[Eu(C_{18}H_{30}O_7N_2Si_2Cl_3+Na)]^+$
3	1211.180	1211.275	$[Eu(C_{38}H_{66}O_{15}N_2Si_4Cl_3+Na)]^+$
4	1321.270	1319.293	$[Eu(C_{42}H_{80}O_{18}N_4Si_4Cl_3+Na)]^+$

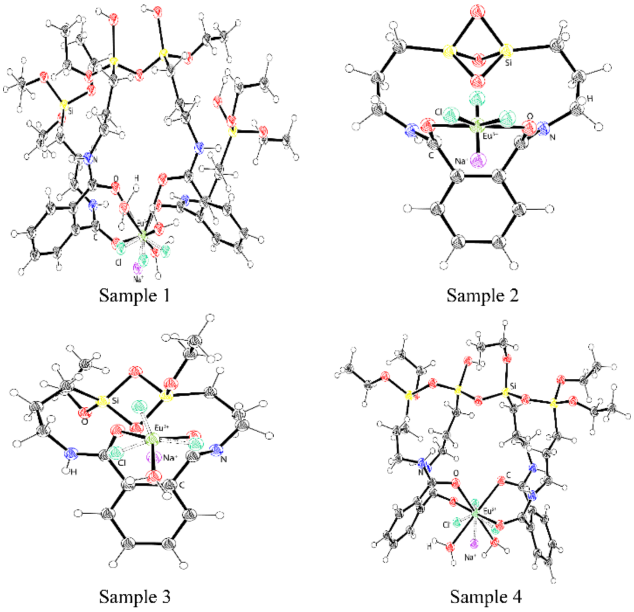

Figure S2. Geometry structures of products
